# Enhanced Visualization: Transforming Non-Contrast into Contrast-Enhanced Computed Tomography Images Through Advanced Generative Adversarial Networks

**DOI:** 10.3390/diagnostics16060861

**Published:** 2026-03-13

**Authors:** Hyun Soo Kim, Bo Mi Gil, Taehwan Kim, Yeo Dong Yoon, Dae Hee Han

**Affiliations:** 1 Department of Radiology, Bucheon St. Mary’s Hospital, College of Medicine, The Catholic University of Korea, Bucheon 14627, Republic of Korea; kohi0506@cmcnu.or.kr; 2 The AI R&D Center, Polestar Healthcare Inc., Seoul 06621, Republic of Korea; taehwan@polestarhc.com (T.K.); david@polestarhc.com (Y.D.Y.); 3 Department of Radiology, Seoul St. Mary’s Hospital, College of Medicine, The Catholic University of Korea, Seoul 06591, Republic of Korea; dhhan@catholic.ac.kr

**Keywords:** generative adversarial network (GAN), lymphoma, non-contrast computed tomography (NCCT), synthetic contrast enhanced computed tomography (sCECT)

## Abstract

**Background/Objectives:** Contrast-enhanced CT (CECT) is essential for mediastinal and lymph node assessment but is often limited in patients with renal dysfunction, prior severe contrast reactions, or pediatric populations. Deep learning approaches, such as generative adversarial networks (GANs), allow the generation of synthetic CECT (sCECT) from non-contrast CT (NCCT) without contrast injection. **Materials and Methods:** A GAN-based model was trained using 400 CECT scans acquired between March and July 2024. The model was tested in 20 patients with lymphoma or metastatic lymphadenopathy diagnosed between January and July 2025, using only NCCT scans. Quantitative evaluation compared sCECT with CECT using Mean Absolute Error (MAE), Root Mean Square Error (RMSE), Peak Signal-to-Noise Ratio (PSNR), Structural Similarity Index (SSIM), and Pearson Correlation Coefficient (PCC). Two radiologists performed qualitative assessment, and Signal-to-Noise Ratio (SNR)/Contrast-to-Noise Ratio (CNR) values were measured for thoracic structures. **Results:** Compared with NCCT, sCECT demonstrated slightly lower MAE (20.87 ± 8.84 vs. 21.26 ± 9.26) and RMSE (45.22 ± 14.22 vs. 45.94 ± 15.07), and marginally higher PSNR (15.44 ± 2.70 vs. 15.38 ± 3.02), indicating modest improvements in pixel-wise similarity. SSIM values were comparable (0.610 ± 0.09 vs. 0.63 ± 0.10), while PCC decreased (0.61 ± 0.09 vs. 0.77 ± 0.15). All differences were statistically significant (*p* < 0.001). Despite these mixed quantitative results, sCECT was qualitatively rated significantly higher by radiologists, with improved visualization of mediastinal structures. SNR and CNR analyses further supported enhanced contrast depiction in sCECT compared with NCCT. **Conclusions:** The GAN-based model successfully generated sCECT from NCCT with modest quantitative similarity gains but clear qualitative improvement, particularly for mediastinal lymph node evaluation. Although synthetic enhancement represents a learned intensity transformation rather than true iodine-based attenuation, sCECT may serve as a valuable adjunct in patients with contraindications to iodinated contrast.

## 1. Introduction

Iodinated contrast media are extensively utilized in computed tomography (CT) to enhance tissue contrast, allowing for better visualization of anatomical structures and pathological conditions [1,2]. However, iodinated contrast media are associated with potential risks, ranging from mild physiological responses to severe, life-threatening complications [3,4]. As a result, many chest CT scans—particularly those performed for screening or initial evaluations—are conducted without contrast enhancement. While non-contrast CT (NCCT) is generally sufficient for assessing pulmonary parenchymal diseases, alternative approaches such as contrast agent substitution after allergy evaluation or the use of other imaging modalities including magnetic imaging (MRI), may be considered in selected patients. However, contrast-enhanced CT (CECT) remains the most widely used modality for accurate evaluation of the mediastinum, pleura, and thoracic vasculature.

For the diagnosis and staging of lymphoma, CT remains the most used modality. NCCT, however, often makes it challenging to delineate lymph nodes from adjacent anatomical structures, complicating the distinction between enlarged lymph nodes and normal tissues. Therefore, accurate measurement of lymph node size and features typically requires the use of CECT [5]. Nevertheless, the use of contrast agents may be contraindicated or limited in patients with impaired renal function, pediatric populations, or those with a prior history of severe contrast-induced adverse reactions.

In recent years, deep learning has gained significant attention in medical imaging, particularly the use of generative adversarial networks (GAN) to synthesize images [6,7,8,9,10]. These approaches have received significant recognition for their ability to produce highly realistic and detailed medical images [6]. Several studies have explored the generation of synthetic contrast-enhanced CT (sCECT) images from NCCT. However, to our knowledge, limited research has addressed the clinical applicability of these synthetic images in the diagnostic assessment of thoracic diseases. Therefore, we aimed to generate sCECT images from NCCT using advanced GAN-based deep learning model and assess their diagnostic value on detecting mediastinal lymph nodes, particularly in patients with lymphoma or other lymphadenopathy (LAP).

## 2. Materials and Methods

This retrospective observational cohort study was approved by the institutional review board (IRB) of our institution (IRB number: HC24RISI0014). The requirement for informed consent was waived due to the retrospective nature of the study.

### 2.1. Study Participants

**Figure 1:**
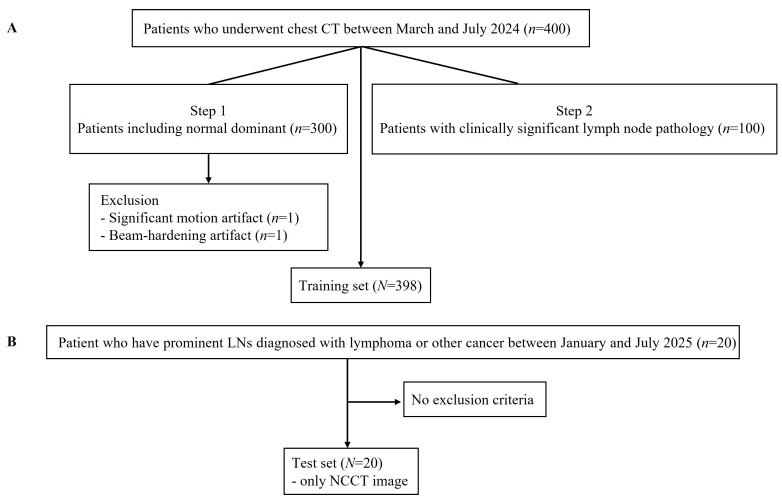
Patient selection flow chart showing inclusion and exclusion for training set (**A**) and test set (**B**).

Patient selection flow chart are shown in Figure 1. For model development, a total of 400 consecutive CECT scans acquired between March and July 2024 were collected.

The training dataset was constructed in two steps: (1) 300 consecutive patients who underwent chest CECT during the study period were included. These patients presented with a spectrum of lymph node findings, including normal lymph nodes, reactive lymphadenopathy, metastatic lymph nodes, sarcoidosis, or indeterminate lymph nodes. (2) an additional 100 patients diagnosed with lymphoma or other lymphadenopathy (LAP) were separately identified and included to enrich the dataset with clinically significant lymph node pathology. Among the 400 collected cases, two patients were excluded due to motion artifact (*n* = 1) or beam hardening artifact (*n* = 1), resulting in a final training set of 398 patients (298 patients including normal dominant findings and 100 patients with lymphoma or LAP).

For independent evaluation, a separate test set, 20 patients with prominent lymph nodes diagnosed with lymphoma or other malignancies between January and July 2025 were included.

Data splitting was performed strictly at the patient level to prevent data leakage. In addition, the test cohort was temporally separated from the training cohort (2025 vs. 2024), and patient identifiers were cross-checked to ensure that no individual patient was included in both datasets.

All patients underwent both NCCT and CECT. Only the NCCT images were used as input to generate sCECT images, while the corresponding real CECT images served as the reference standard for comparison. All cases had acceptable image quality and were free of major artifacts. This design enabled objective evaluation of the generated sCECT images against CECT data for both technical similarity and clinical usefulness.

### 2.2. CT Technique and Image Processing

All chest CT examinations were performed in the supine position using a 64-slice multi-detector CT scanner (SOMATOM Sensation 64; Siemens Healthcare, Erlangen, Germany). Scanning was conducted using a fixed tube voltage of 120 kVp, with tube current modulation applied (reference range: 60–120 mAs) according to patient size. The gantry rotation time was 0.5 s with a pitch of 1.0. Axial images were acquired from the lower neck to the upper abdomen and reconstructed with a slice thickness between 1.5 and 2.5 mm using a standard soft tissue kernel. Images were reviewed using mediastinal window settings (window width: 450 Hounsfield units [HU]; window level: 60 HU) for consistency in both training and evaluation stages. All images were exported in Digital Imaging and Communications in Medicine (DICOM) format after anonymization. Pixel values were normalized to the range −1 to 1 before being input into a deep learning model. The generation of sCECT images from NCCT was performed using a GAN-based model, as described in the following subsection.

### 2.3. Model Architecture and Training

We used the CycleGAN framework [11], a member of the GAN family, to train on patient-matched pre-processed NCCT-CECT data acquired in the same examination session, without additional spatial registration. Two generators translated NCCT to CECT and vice versa, whereas two discriminators were trained to distinguish the synthetic images produced by each generator from real images. NCCT and CECT images were acquired during the same examination session using identical reconstruction parameters. Slice was performed within each patient based on corresponding anatomical positions, but no additional rigid or deformable registration was applied between NCCT and CECT. Therefore, despite same-session acquisition, residual spatial misalignment may remain due to respiration, subtle motion, and contrast enhancement timing differences. Accordingly, we adopted CycleGAN to mitigate the need for perfectly registered voxel-wise pairs during training.

**Figure 2:**
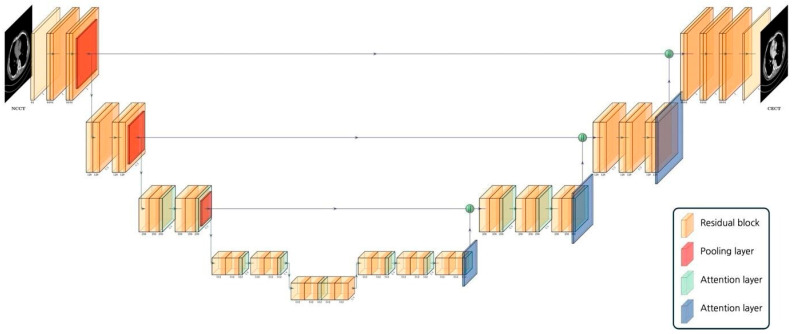
Architecture of the generator for NCCT-to-CECT translation. CECT = contrast-enhanced CT; NCCT = non-contrast CT.

Both generators adopted an Attention-UNet architecture [12], as illustrated in Figure 2. The network begins with a 7 × 7 reflection-padded convolution with 64 filters and follows an encoder–decoder layout in which spatial resolution is progressively halved while the number of channels doubles. Each residual block consists of two sequential 3 × 3 convolutions with intermediate normalization and activation, and it adds the output back to the original input or, when the channel width differs, to its 1 × 1. Residual blocks are combined with a self-attention module [13] that captures long-range contextual information essential for recovering subtle parenchymal contrast in the synthetic CECT. Decoder blocks uP-sample the features and receive skip connections from the corresponding encoder layers, and a final 3 × 3 convolution with tanh activation yields an intensity-normalized single-channel image.

The two discriminators are PatchGAN [11] classifiers that incorporate self-attention after the second and third convolutional layers; each consists of four consecutive 4 × 4 stride-two convolutions whose channel widths rise from 64 to 128, 256, and 512, followed by a final 4 × 4 stride-one convolution that outputs a grid of logits. By operating on image patches instead of whole images, they focus on local texture realism, while the attention modules guide the network toward contrast-informative regions.

Let x ∈ X denote an NCCT slice and y ∈ Y its paired CECT slice. Two generators learn the bidirectional mappings G_XY:X→Y and G_YX:Y→X, whereas two discriminators, D_Y and D_X, evaluate the realism of synthetic CECT and NCCT images, respectively. Generator optimization minimizes the objective function,

$$L_{G}=L_{adv}+\lambda_{cyc}L_{cyc}+\lambda_{id}L_{id}+\lambda_{gd}L_{gd}+L_{perceptual}+\lambda_{ssim}L_{ssim}+\lambda_{vox}L_{vox},$$

where the adversarial term,

$$L_{adv}=BCE\left[D_{Y}\left(G_{XY}\left(x\right)\right),\;1\right]+BCE\left[D_{X}\left(G_{YX}\left(y\right)\right),\;1\right],$$

with BCE denoting the binary cross-entropy loss, encourages the synthetic images to be classified as real by their respective discriminators. The cycle-consistency term,

$$L_{cyc}={\left\|G_{YX}\left(G_{XY}\left(x\right)\right)-x\right\|}_{1}+{\left\|G_{XY}\left(G_{YX}\left(y\right)\right)-y\right\|}_{1},$$

forces the two generators to behave as approximate inverses, whereas the identity term,

$$L_{id}={\left\|G_{XY}\left(y\right)-y\right\|}_{1}+{\left\|G_{YX}\left(x\right)-x\right\|}_{1},$$

preserves voxel intensities when the input already belongs to the target domain. Edge fidelity is promoted by the gradient-difference term,

$$L_{gd}={\left\|\nabla G_{XY}\left(x\right)-\nabla y\right\|}_{1}+{\left\|\nabla G_{YX}\left(y\right)-\nabla x\right\|}_{1},$$

while perceptual similarity is enforced through a VGG16-based perceptual loss $L_{perceptual}$. Structural coherence is addressed by

$$L_{ssim}=-loglog\;SSIM\left(G_{XY}\left(x\right),\;y\right)-loglog\;SSIM\left(G_{YX}\left(y\right),\;x\right),$$

and overall intensity agreement by the voxel-wise

$$L_{1}\mathrm{loss}\;L_{vox}={\left\|G_{XY}\left(x\right)-y\right\|}_{1}+\|G_{YX}\left(y\right)-x\|.$$

Throughout training, we set $\lambda_{cyc}$, $\lambda_{id}$, $\lambda_{gd}$, and $\lambda_{vox}$ to 10, and $\lambda_{ssim}$ to 0.5. The discriminators are trained with the complementary objective

$$L_{D}=\frac{1}{2}\left[BCE\left(D_{Y}\left(y\right),1\right)+BCE\left(D_{Y}(G_{XY}(x)\right),\;0)+BCE\left(D_{X}\left(x\right),1\right)+BCE\left(D_{X}\left(G_{YX}(y)\right),\;0\right)\right]$$.

The two generators and both discriminators were trained with their respective objectives, $L_{G}$ for the generators and $L_{D}$ for the discriminators. In our experiments, after training, we evaluated only the NCCT-to-CECT generator $G_{XY}$, because the clinical use-case of interest is to synthesize contrast-enhanced images from non-contrast CT scans. The reverse-direction generator, $G_{YX}$, and both discriminators were required to optimize $G_{XY}$ during training and were discarded during the inference phase.

### 2.4. Image Analysis—Quantitative

Quantitative image similarity was assessed by sCECT and NCCT images against the ground truth CECT. The evaluation metrics included mean absolute error (MAE), root mean square error (RMSE), peak signal-to-noise ratio (PSNR), structural similarity index (SSIM), and Pearson correlation coefficient (PCC). Lower values of MAE and RMSE, as well as higher values of PSNR and SSIM, and a PCC closer to 1, indicate a greater resemblance between NCCT or sCECT and CECT images [14,15]. Whereas RMSE and PSNR quantify absolute errors, SSIM evaluates image similarity from a perception-based perspective. Therefore, SSIM is generally regarded as a more appropriate metric than RMSE and PSNR for assessing image similarity in terms of human visual perception [16]. Additionally, regions of interest were manually placed to measure signal-to-noise ratio (SNR) and contrast-to-noise ratio (CNR) in the ascending aorta (AA), main pulmonary artery (MPA), and lymph nodes (LNs) for comparing contrast visualization. SNR and CNR measurements were independently performed by each reviewer. Statistical analyses were conducted separately for each reviewer, and results were reported individually rather than combined.

### 2.5. Image Analysis—Qualitative

For the qualitative analysis, two radiologists independently reviewed NCCT, sCECT, and CECT images in random order. One reviewer was a radiology resident with 2 years of clinical experience, and the other was a board-certified thoracic radiologist with 12 years of experience, including 4 years of residency and 8 years as a practicing specialist. Reviewers were blinded to the image type and clinical information. They rated each scan using a 3-point scale based on anatomic clarity and vascular enhancement: (1) poor (obscured anatomic detail, enhancement insufficient for diagnosis), (2) moderate (clear anatomic detail with partial or uneven vascular enhancement), and (3) excellent (distinct anatomic detail with nearly complete uniform vascular enhancement). Furthermore, for the test set of 20 cases, overall diagnosis (lymphoma vs. metastatic lymph node) was made independently using both CECT and sCECT. Inter-observer agreement was compared between the methods.

### 2.6. Statistical Analysis

Statistical analyses were performed using statistical software (R studio version 4.0.3; R Foundation for Statistical Computing, Vienna, Austria). For comparison of quantitative metrics, normality was assessed using the Kolmogorov–Smirnov test. Depending on the distribution, paired *t*-tests were applied for normally distributed data, and the Wilcoxon signed-rank test was used for non-normally distributed data. Continuous variables such as SNR and CNR were expressed as mean ± standard deviation. For qualitative analysis, categorical variables were compared using the chi-square test. Interobserver agreement for qualitative scores was evaluated using Cohen’s κ statistics. A *p*-value < 0.05 was considered statistically significant.

## 3. Results

It should provide a concise and precise description of the experimental results, their interpretation, as well as the experimental conclusions that can be drawn.

### 3.1. Patient Characteristics

Patient characteristics are summarized in Table 1. In the training set (*n* = 298), the median age was 62 years (interquartile range [IQR], 55–69 years) with 109 men and 189 women. Of these, 257 patients (86.2%) had normal lymph nodes, 33 (11.1%) had reactive lymphadenopathy, 6 (2.0%) had metastatic lymph nodes, 1 (0.3%) had sarcoidosis-related lymphadenopathy, and 1 (0.3%) had indeterminate lymph node. Among the additional 100 patients with suspected or diagnosed with lymphoma or other LAP, the median age was 70.5 years (IQR, 57–81 years), with 59 men and 41 women. For the test set (*n* = 20), the median age of the 20 patients was 71 years (IQR, 60–80 years), with 11 men and 9 women. Sixteen patients (80.0%) had lymphoma, and the remaining cases included thymic carcinoma (*n* = 1, 5.0%), lung cancer (*n* = 1, 5.0%), advanced gastric cancer (*n* = 1, 5.0%), and pancreatic cancer (*n* = 1, 5.0%).

### 3.2. Quantitative Analysis

Quantitative image similarity results are summarized in Table 2. Compared with NCCT, sCECT demonstrated slightly lower MAE (20.87 vs. 21.26) and RMSE (45.22 vs. 45.94), and marginally higher PSNR (15.44 vs. 15.38), indicating modest improvement in pixel-wise similarity to CECT. SSIM values were comparable between sCECT and NCCT (0.609 vs. 0.627), whereas PCC was lower for sCECT (0.609) compared with NCCT (0.770), indicating somewhat weaker intensity alignment. All differences were statistically significant (*p*-value < 0.001).

**Figure 3:**
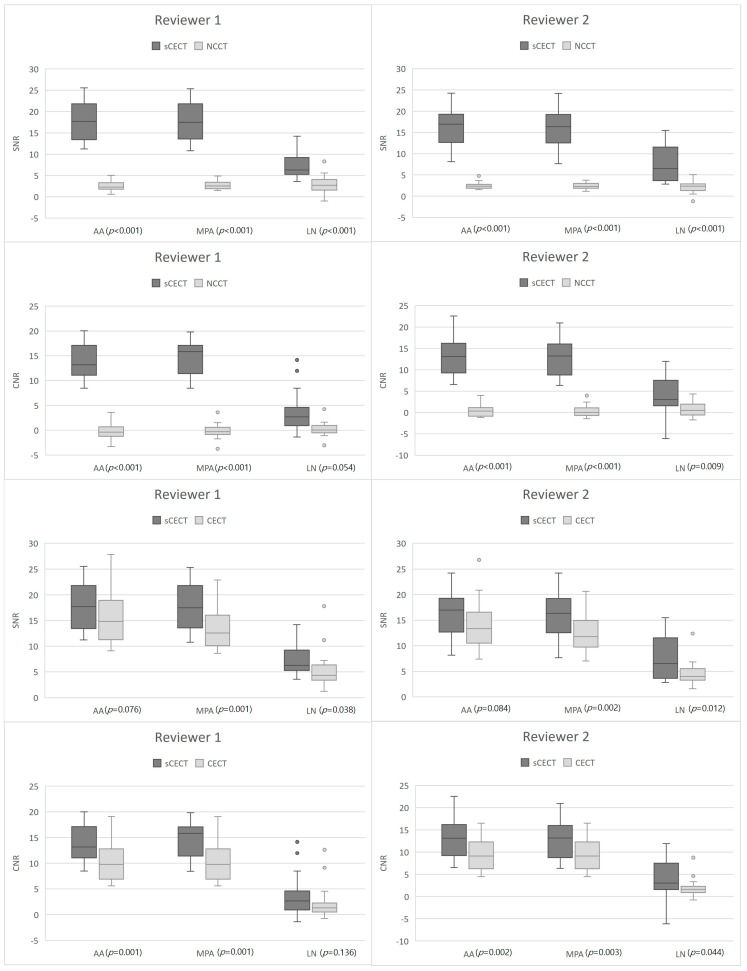
Comparison of SNR and CNR values of NCCT, sCECT, and CECT as measured by Reviewer 1 and 2 in the AA, MPA, and LN. Note Data are median with interquartile range. AA = ascending aorta; CECT = contrast-enhanced CT; CNR = contrast-to-noise ratio; LN = lymph node; MPA = main pulmonary artery; NCCT = non-contrast CT; sCECT = synthetic contrast-enhanced CT; SNR = signal-to-noise ratio.

Figure 3 shows the box plots comparing SNR and CNR values among NCCT, sCECT, and CECT. In the comparison between NCCT and sCECT, both reviewers observed significantly higher SNR and CNR values of sCECT across all anatomical regions (AA, MPA, LN, all *p* < 0.05), except for the LN CNR measured by reviewer 1, which did not reach statistical significance (*p* = 0.054). When comparing sCECT with CECT, SNR and CNR values were generally higher for sCECT in all anatomical regions. However, no statistically significant differences were found for AA SNR (reviewer 1: *p* = 0.076, reviewer 2: *p* = 0.084) and LN CNR (reviewer 1: *p* = 0.136), suggesting that sCECT achieved comparable contrast conspicuity to CECT in these regions.

### 3.3. Qualitative Analysis

Both reviewers consistently rated sCECT significantly higher than NCCT (*p* < 0.001) in anatomic clarity and vascular enhancement. For one reader, the combination of NCCT plus sCECT achieved image quality comparable to CECT, with no statistically significant difference (*p* = 0.1712). Furthermore, sCECT facilitated better visualization of abnormal mediastinal lymph nodes, contributing to improved diagnostic confidence. Interobserver agreement for overall diagnostic decisions in the test set was excellent (Cohen’s kappa = 0.894, *p* < 0.001). Diagnostic agreement between sCECT-based and CECT-based assessments was 85% (17/20 cases), with only one discordant case in the small test cohort.

## 4. Discussion

CECT plays a crucial role in the assessment of mediastinal structures and lymph nodes [1,2]. However, its use can be challenging in certain patient populations, such as those with chronic kidney disease, prior severe contrast reactions, or pediatric patients for whom repeated contrast administration is undesirable. Deep learning approaches, such as GAN-based models, now allow generation of sCECT from NCCT without contrast injection. In this study, we applied this technique to mediastinal lymph node assessment in patients with lymphoma to evaluate its diagnostic utility.

The study aimed to generate sCECT images from NCCT using an advanced GAN-based deep learning model and evaluate their image quality and diagnostic value for mediastinal lymph node assessment. sCECT achieved lower MAE, RMSE and higher PSNR compared with NCCT, indicating closer similarity to CECT.

In the quantitative analysis, although sCECT demonstrated statistically significant improvements in MAE, RMSE, and PSNR compared with NCCT, the magnitude of these changes was modest. This likely reflects the fact that global pixel-wise metrics are influenced by the entire image volume, the majority of which consists of lung parenchyma, soft tissue, and background regions that show minimal enhancement differences between NCCT and CECT. Because contrast enhancement affects primarily vascular structures and lymph nodes, its impact on whole-image similarity metrics may be diluted.

Interestingly, SSIM values were slightly lower for sCECT compared with NCCT, and PCC showed a more noticeable reduction. PCC reflects voxel-wise intensity correlation with CECT across the entire image. However, contrast enhancement primarily affects specific anatomical structures such as vessels and lymph nodes rather than the whole image volume. Because sCECT selectively enhances these regions, the overall voxel-wise intensity correlation with CECT may not increase uniformly across all pixels. Therefore, a reduced PCC does not necessarily indicate poorer depiction of clinically relevant structures.

These findings suggest that conventional global similarity metrics may not fully capture clinically meaningful improvements in regional contrast enhancement. This interpretation is supported by the significantly higher SNR and CNR values observed in vascular structures and lymph nodes, as well as the improved qualitative ratings by both reviewers. Therefore, while voxel-level intensity correlation was not uniformly increased, sCECT appears to enhance diagnostically relevant contrast features in a manner that improves subjective image quality and lesion conspicuity.

Moreover, sCECT images demonstrated significantly higher SNR and CNR values than NCCT across the anatomical regions (AA, MPA, LN) for both reviewers (*p* < 0.05), except for the LN CNR measured by reviewer 1, which narrowly missed statistical significance (*p* = 0.054). Importantly, in AA, sCECT demonstrated values that were nearly indistinguishable from those of CECT, suggesting that enhancement of large central vessels—where bolus arrival is relatively consistent—is well replicated by the synthetic model. This finding is of value because aortic enhancement often serves as the reference threshold for initiating image acquisition in routine CECT protocols, and its stability may allow deep learning models to reproduce enhancement patterns more faithfully. In contrast, the MPA exhibited greater variability between sCECT and CECT, as observed by both reviewers. This discrepancy is likely attributable to the complex hemodynamic characteristics of the pulmonary circulation. Unlike the aorta, pulmonary arterial enhancement is more susceptible to interpatient variability in cardiac output, vessel diameter, and timing of bolus arrival, all of which can introduce heterogeneity in enhancement patterns on CECT. Such dynamic physiologic variation is difficult for GAN-based reconstruction to replicate, and thus may explain the relative inconsistency in SNR and CNR values for the MPA. For mediastinal lymph nodes, sCECT achieved CNR values approaching those of CECT, with one reviewer finding no significant difference. This observation is clinically meaningful, as accurate delineation of lymph nodes from adjacent mediastinal structures is critical in the staging and follow-up of lymphoma. By narrowing the performance gap with CECT in this domain, sCECT demonstrates the potential to serve as a useful adjunct for diagnostic decision-making, especially in patients for whom iodinated contrast is contraindicated. Nevertheless, sCECT cannot fully reproduce the dynamic hemodynamic changes observed with contrast administration [17,18,19]. Importantly, synthetic contrast enhancement represents an intensity transformation learned from imaging data rather than true iodine-based attenuation changes. Therefore, while sCECT may improve visual contrast conspicuity, it does not reflect actual contrast material distribution or quantitative attenuation values. Caution is warranted when considering applications that rely on precise vascular enhancement or quantitative Hounsfield unit measurements. Consequently, while sCECT can serve as a valuable adjunct or partial substitute for CECT—particularly when anatomical delineation is critical—it may not entirely replace CECT in clinical scenarios requiring hemodynamic information.

From a practical clinical perspective, sCECT may be particularly useful in patients for whom contrast administration is contraindicated or undesirable, such as those with renal dysfunction, prior severe contrast reactions, pediatric patients, or patients requiring repeated imaging. In such settings, sCECT could be integrated into routine NCCT workflows to provide additional contrast information without increasing scan time or requiring contrast administration. Furthermore, it may be useful as a complementary tool to improve lesion conspicuity and diagnostic confidence, particularly in the evaluation of mediastinal lymph nodes.

In qualitative analysis, both reviewers assigned significantly higher scores to sCECT compared to NCCT (*p* < 0.001). For one reviewer, the combined use of NCCT and sCECT achieved image quality equivalent to CECT (*p* < 0.1712). Notably, sCECT facilitated the detection of mediastinal lymph nodes, proving particularly useful in lymphoma patients with prominent mediastinal lymphadenopathy, and interobserver agreement was excellent. Although a 3-point scale was used for image quality assessment, this simplified grading system was selected to enhance reproducibility and minimize interobserver variability. The excellent interobserver agreement (κ = 0.894) suggests that this scale provided sufficient discriminatory ability for the purposes of this study. Nevertheless, the limited granularity of a 3-point scale may introduce potential ceiling effects, and future studies using more detailed scoring systems could provide further refinement. Notably, diagnostic agreement between sCECT and CECT reached 85% in the test cohort, further supporting the clinical relevance of the qualitative improvements observed.

**Figure 4:**
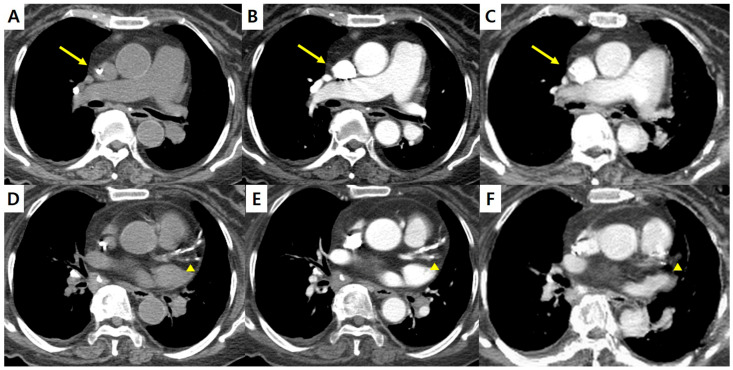
Chest CT images of a 76-year-old woman with lymphoma. (**A**,**D**) NCCT, (**B**,**E**) CECT, and (**C**,**F**) sCECT. sCECT demonstrates reduced beam-hardening artifacts caused by medical devices in the superior vena cava (arrows). Coronary artery calcifications (arrowheads) appear less conspicuous on sCECT compared with NCCT and CECT. CECT = contrast-enhanced CT; NCCT = non-contrast CT; sCECT = synthetic contrast-enhanced CT.

**Figure 5:**
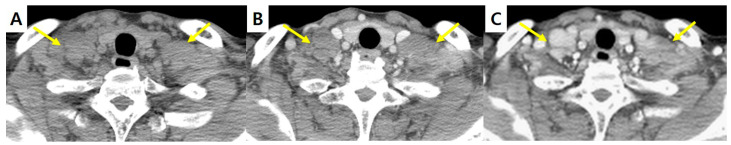
Neck CT images of a 59-year-old man with lymphoma. (**A**) NCCT, (**B**) CECT, (**C**) sCECT. sCECT provides clearer differentiation between neck muscles and lymph nodes (arrows) compared with NCCT, allowing improved anatomical visualization. CECT = contrast-enhanced CT; NCCT = non-contrast CT; sCECT = synthetic contrast-enhanced CT.

**Figure 6:**
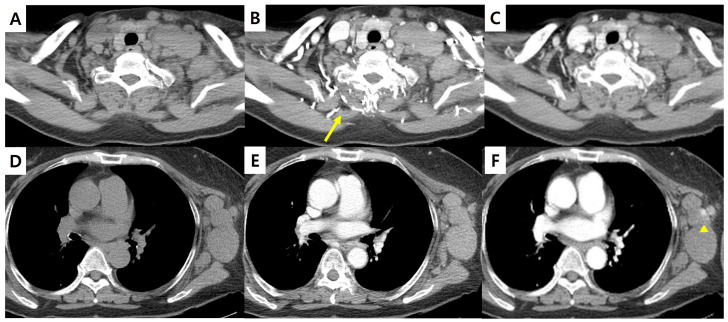
Chest CT images of a 64-year-old woman with lymphoma. (**A**,**D**) NCCT, (**B**,**E**) CECT, and (**C**,**F**) sCECT. Flow artifact from collateral vessel contrast filling (arrow) is prominent on CECT but markedly reduced on sCECT. Mild over-enhancement of non-specific areas, such as the left axilla (arrowhead), was occasionally observed. CECT = contrast-enhanced CT; NCCT = non-contrast CT; sCECT = synthetic contrast-enhanced CT.

Representative cases from the test set are shown in Figure 4, Figure 5 and Figure 6. In Figure 4, sCECT demonstrates reduced beam-hardening artifacts from a central venous catheter compared with NCCT and CECT and improves overall mediastinal visualization. However, coronary artery calcifications appear less conspicuous on sCECT, which may be a limitation in cases where calcification burden assessment is required. Figure 5 highlights the superior differentiation of cervical lymph nodes and adjacent muscles on sCECT relative to NCCT, improving lesion conspicuity. Additionally, in Figure 6, sCECT shows marked reduction in contrast-related flow artifacts seen on CECT, resulting in cleaner depiction of vascular structures. In clinical practice, CECT images are acquired after intravenous contrast administration with an inherent time delay, which often results in misalignment between NCCT and CECT images due to respiratory motion, bowel peristalsis, or patient repositioning [20,21,22]. Because sCECT is generated directly from NCCT data, it eliminates this temporal gap and provides anatomically matched images, allowing more reliable one-to-one comparison at the same anatomical level.

Recently, studies have focused on synthesizing MRI from CT, generating missing MRI sequences [23], or creating sCECT from non-enhanced CT (NECT). Lyu et al. reported the potential of producing synthetic CT angiographic images of the neck and abdomen without the use of iodinated contrast, showing promising outcomes in terms of both quantitative and qualitative image quality, as well as diagnostic accuracy, when compared with conventional CT angiograph [24]. In thoracic imaging, Choi et al. demonstrated that deep learning methods based on GANs achieve excellent performance in image similarity metrics and significantly enhance visualization of mediastinal lymph nodes when generating sCECT images from NCCT [25]. Jeon et al. reported that combining a Residual U-net with a Convolutional Block Attention Module significantly enhanced the detection of hilar lymph nodes on virtual contrast-enhanced CT reconstructed from NCCT [26]. However, to our knowledge, research on the clinical utility of sCECT for actual thoracic disease diagnosis remains limited. Therefore, our study carries significant value in that it applies synthetic image conversion technology to thoracic imaging, thereby demonstrating its potential contribution to the advancement of diagnostic imaging research.

## 5. Limitation

This study has several limitations. First, the test cohort included a relatively small number of lymphoma or advanced cancer patients. Although the training set encompassed patients with suspected or confirmed lymphoproliferative disorders, validation in larger and more diverse cohorts is needed to confirm the robustness of the findings. Second, sCECT occasionally produced unnecessary or insufficient enhancement in certain regions, such as the axilla in Figure 6F. These discrepancies may reflect limitations in the training dataset and the model architecture, which could further improve performance in clinical application. Third, all CT examinations were acquired using a single scanner model with consistent acquisition and reconstruction parameters. While this ensured internal consistency and minimized technical variability during model development, it may limit the generalizability of the model to images obtained from different scanner vendors or imaging protocols. Variations in reconstruction kernels, noise characteristics, and contrast timing across institutions could influence model performance. Therefore, future multi-center and multi-vendor validation studies are warranted to establish broader clinical applicability.

## 6. Conclusions

In conclusion, this study demonstrated that sCECT generated from NCCT using a GAN-based model improved image quality for detection of abnormal mediastinal lymph nodes, suggesting potential clinical utility in patients with lymphoma or advanced cancer. Although current techniques cannot fully replicate the hemodynamic effects of contrast administration, they may offer a valuable alternative in an aging population with increasing chronic kidney disease, contrast allergy, or pediatric patients where contrast use is limited.

## Figures and Tables

**Table 1:** Patient characteristics. Percentages may not sum to 100% due to rounding.

Patient Characteristics					
	Training Set (*n* = 298)		Training Set (*n* = 100)	Test Set (*n* = 20)	
Age (y)	62 (55–69)		70.5 (57–81)	71 (60–80)	
Sex (male/female)	109/189		59/41	11/9	
Clinical indication	Normal	257(86.2%)	Lymphoma or Other LAP	Lymphoma	16 (80.0%)
	Reactive LN	33(11.1%)		Thymic carcinoma	1 (5.0%)
	Metastatic LN	6 (2.0%)		Lung cancer	1 (5.0%)
	Sarcoidosis	1 (0.3%)		Advanced gastric cancer	1 (5.0%)
	Indeterminate LN	1 (0.3%)		Pancreatic cancer	1 (5.0%)

LAP = lymphadenopathy; LN = lymph node.

**Table 2:** Comparison of the mean similarity metric values between non-contrast CT (NCCT) and synthetic contrast-enhanced CT (sCECT), using contrast-enhanced CT as the reference standard. Lower MAE, lower RMSE, higher PSNR, higher SSIM, and PCC closer to 1 indicate higher image similarity.

	MAE ↓	RMSE ↓	PSNR ↑	SSIM ↑	PCC
NCCT vs. CECT	21.26 ± 9.26	45.94 ± 15.07	15.38 ± 3.02	0.63 ± 0.10	0.77 ± 0.15
sCECT vs. CECT	20.87 ± 8.84	45.22 ± 14.22	15.44 ± 2.70	0.61 ± 0.09	0.610 ± 0.09

MAE = mean absolute error; PCC = pearson correlation coefficient; PSNR = peak signal-to-noise ratio; RMSE = root mean square error; SSIM = structural similarity index measurement, all *p*-values < 0.001. ↑ Increase; ↓ decrease.

## Data Availability

The raw data supporting the conclusions of this article are not publicly available due to ethical and patient privacy restrictions but are available from the corresponding author upon reasonable request.

## Abbreviations

The following abbreviations are used in this manuscript:

AA	Ascending aorta
CECT	Contrast Enhanced Computed Tomography
CNR	Contrast-to-Noise Ratio
GAN	Generative Adversarial Network
LAP	Lymphadenopathy
LN	Lymph node
MAE	Mean Absolute Error
MPA	Main pulmonary artery
NCCT	Non-Contrast Computed Tomography
PCC	Pearson Correlation Coefficient
PSNR	Peak Signal-to-Noise Ratio
RMSE	Root Mean Square Error
sCECT	Synthetic Contrast Enhanced Computed Tomography
SNR	Signal-to-Noise Ratio
SSIM	Structural Similarity Index
